# Spontaneous Subcutaneous Emphysema in a Teenage Male Extending As Pneumomediastinum, Pneumothorax, Pneumopericardium, and Epidural Pneumatosis: A Rare Combination of Anatomical Locations

**DOI:** 10.7759/cureus.43462

**Published:** 2023-08-14

**Authors:** Lagitha S Selvanayagam, Aruna S Pallewatte, Sivapalan Sivansuthan

**Affiliations:** 1 Internal Medicine, Post Graduate Institute of Medicine, University of Colombo, Colombo, LKA; 2 Radiology, National Hospital of Sri Lanka, Colombo, LKA; 3 General Medicine, Jaffna Teaching Hospital, Jaffna, LKA

**Keywords:** epidural pneumatosis, pneumopericardium, pneumothorax, pneumomediastinum, spontaneous subcutaneous emphysema

## Abstract

Subcutaneous emphysema (SE) and pneumomediastinum can be spontaneous or traumatic in origin. Spontaneous SE involving cervical, parapharyngeal, mediastinal, pericardial, and pleural space together is rare, while epidural pneumatosis is an even rarer entity. We report a previously healthy teenage male with sudden onset chest pain whose plain radiographs and high-resolution computed tomography (HRCT) showed extensive spread of air in the mediastinum, pericardial space, pleural space, and epidural space. He was hemodynamically stable and had a spontaneous recovery after one week. Follow-up radiological imaging showed complete radiological resolution of gas lucencies.

It is quite important for clinicians to be aware of this condition, common and rare routes of extension, and possible complications. Clinical suspicion is vital to plan appropriate investigations especially radiological modalities such as chest X-ray and HRCT. This will help in evaluating the severity of the condition, exclude possible etiologies, and look for potential complications so that proper management and follow-up can be planned.

## Introduction

The term “Emphysema” originates from Greek and Latin and refers to the abnormal accumulation of air in tissues like lungs and limited spaces such as the neck, subcutaneous tissue, and mediastinum [1]. Mediastinal emphysema is also called pneumomediastinum. This was first described by Rene’ Laennec, as a medical condition following trauma in 1819 while spontaneous pneumomediastinum (SPM) is combined with subcutaneous emphysema (SE), it is referred to as Hamman's syndrome, a rare condition first described by Louis Virgil Hamman in 1939 [2].

SE and pneumomediastinum can be divided into spontaneous or traumatic depending on the etiology [3]. Common post-traumatic causes are blunt or penetrating trauma to the chest, iatrogenic invasive interventions [4], barotrauma following mechanical ventilation, etc. [3,5]. Spontaneous SE (SSE) and SPM are classified as primary, when there is no underlying lung disease, and secondary if there is an underlying airway disease, such as cystic fibrosis or asthma [3].

Occurrence of SE in the subcutaneous, mediastinal [6,7], cervical [8], retropharyngeal [9], and parapharyngeal spaces together is a rare phenomenon according to the literature. The presence of air in the epidural space or epidural pneumatosis is an extremely rare entity. Here we report such a unique case of spontaneous primary emphysema having many possible extensions into subcutaneous, retropharyngeal, and parapharyngeal regions with pneumomediastinum, pneumopericardium, pneumothorax, and epidural pneumatosis.

## Case presentation

A 14-year-old previously healthy male presented to us with a sudden onset of chest pain which had started five hours prior to the admission. This pain was a retrosternal, continuous stabbing type, radiating to the neck which increased with lying down position and deep inspiration. The patient had a history of childhood seasonal asthma though he was absolutely free of symptoms for more than five years without any medications including inhalers. There was no fever, cough, vomiting, choking attacks, odynophagia, or dysphagia prior to the presentation. He also denied any trauma, previous surgeries, or inhalation of illicit drugs.

On examination, the patient was not dyspneic, well looking, sitting comfortably on the bed. There was tenderness in the anterior neck and upper chest with crepitations under the skin. Trachea was in the midline with no palpable masses or enlarged lymph nodes. His vital parameters were within normal range with BP of 110/70 mm Hg, PR of 92bpm, and oxygen saturation of 99% on room air. The rest of the systems examinations including respiratory and cardiovascular were unremarkable.

**Figure 1:**
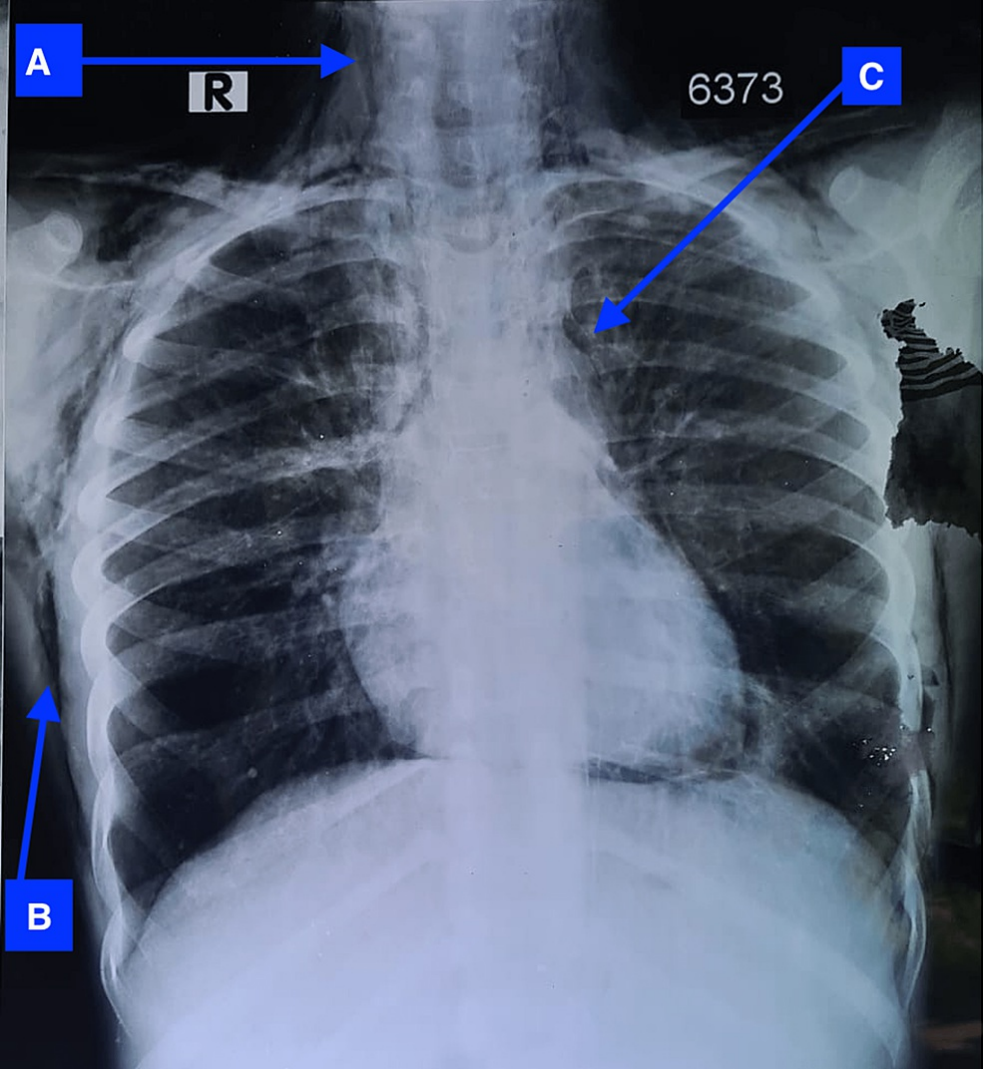
Chest radiograph at the time of initial presentation There is air in neck (A), subcutaneous tissues (B) and mediastinum (C)

**Figure 2:**
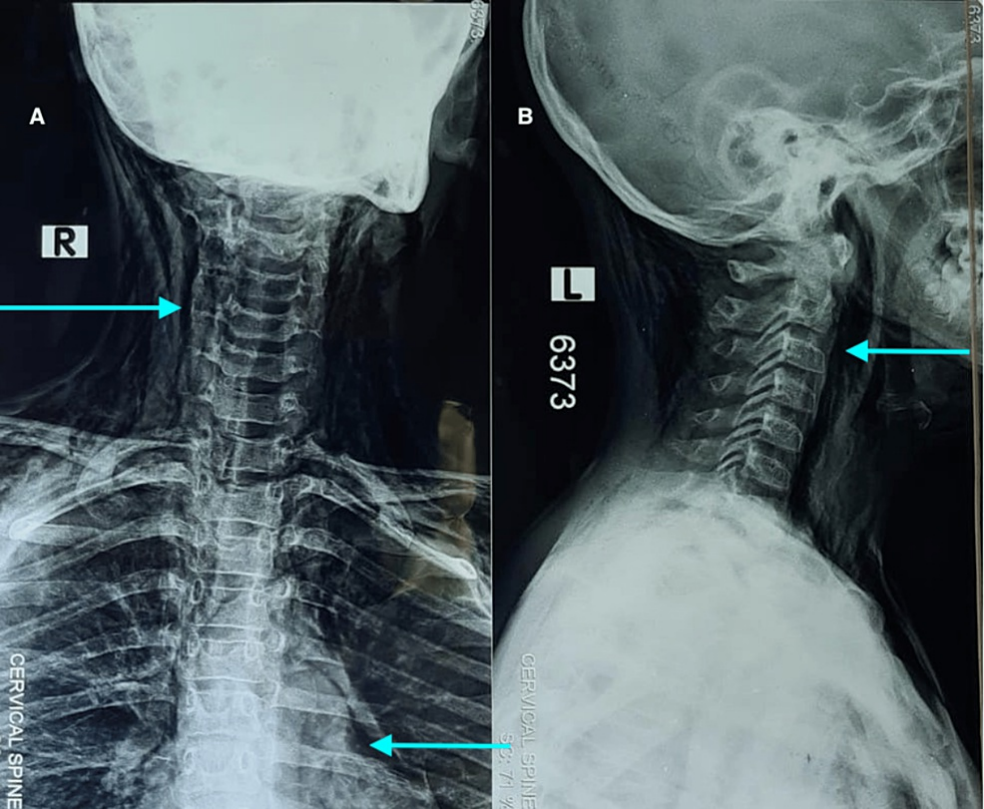
Radiograph of the neck AP (A) and lateral (B) Note that air within the soft tissue planes of the neck (upper arrow in A), pneumomediastinum (lower arrow in A), and parapharyngeal air (arrow in B)

Basic investigations such as ECG, full blood count, inflammatory markers, and renal and liver profile were within normal range. COVID-19 Rapid Antigen Test and COVID-19 PCR were negative. Chest x-ray posteroanterior (PA) view and x-ray neck lateral view showed subcutaneous air on either side of the neck, parapharyngeal space, and in the mediastinum (Figures 1, 2A, 2B).

**Figure 3:**
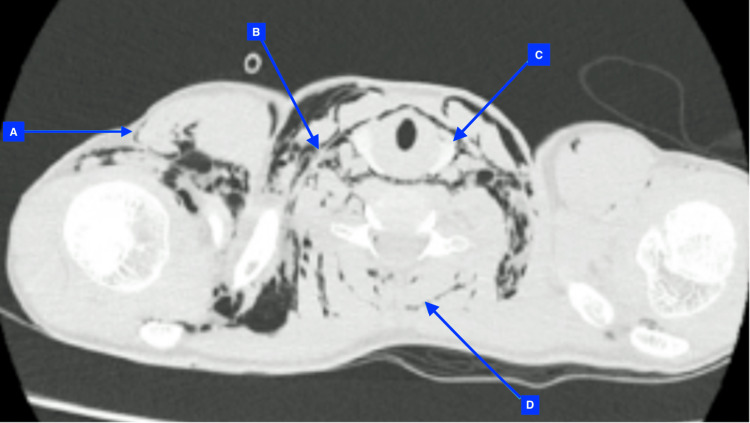
HRCT axial sections through upper chest and neck Note subcutaneous air (A), intermuscular air (B), paratracheal air (C) and air in between erector spinae muscles (D)

High-resolution computed tomography (HRCT) showed extensive spread of air in between muscle planes in the anterior, lateral and posterior chest walls such as pectoralis major, pectoralis minor, serratus anterior, latissimus dorsi, trapezius and superficial intercostal muscles. Emphysema was seen in subcutaneous tissues and fascia. Posteriorly air was seen between erector spinae muscles. These air pockets were more marked in the upper chest with intermuscular and subcutaneous air in the neck (Figure 3). The paratracheal, retropharyngeal spaces also had significant air collections (Figure 3). There was no significant difference between inspiratory and expiratory HRCTs.

**Figure 4:**
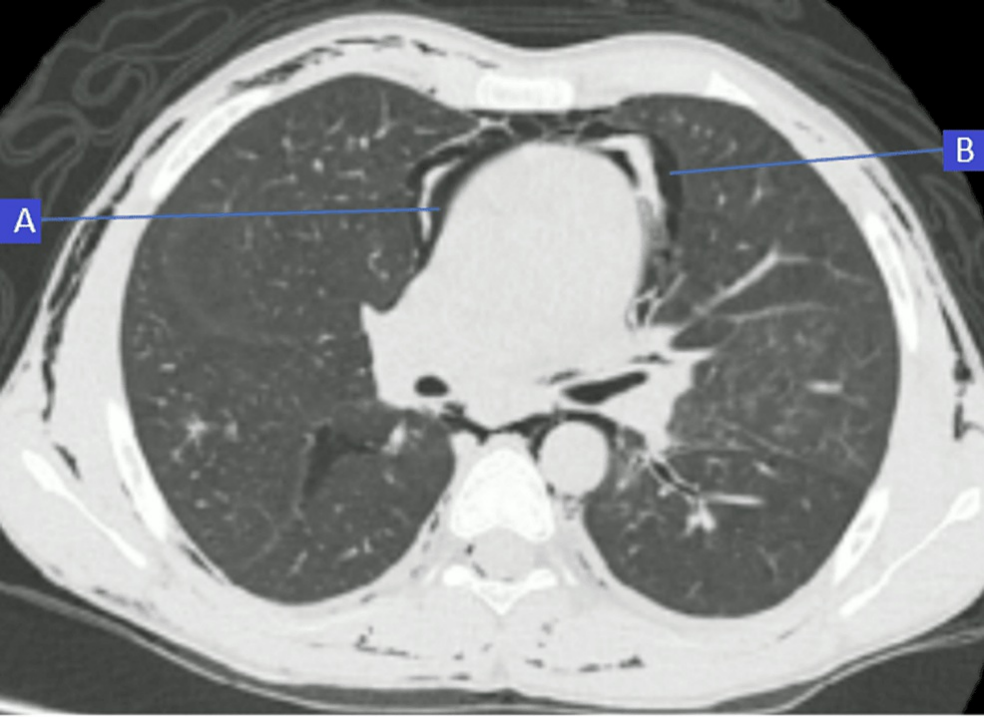
HRCT axial image below the level of carina Pneumopericadium (arrow A) and pnemomediastinum (arrow B)

**Figure 5:**
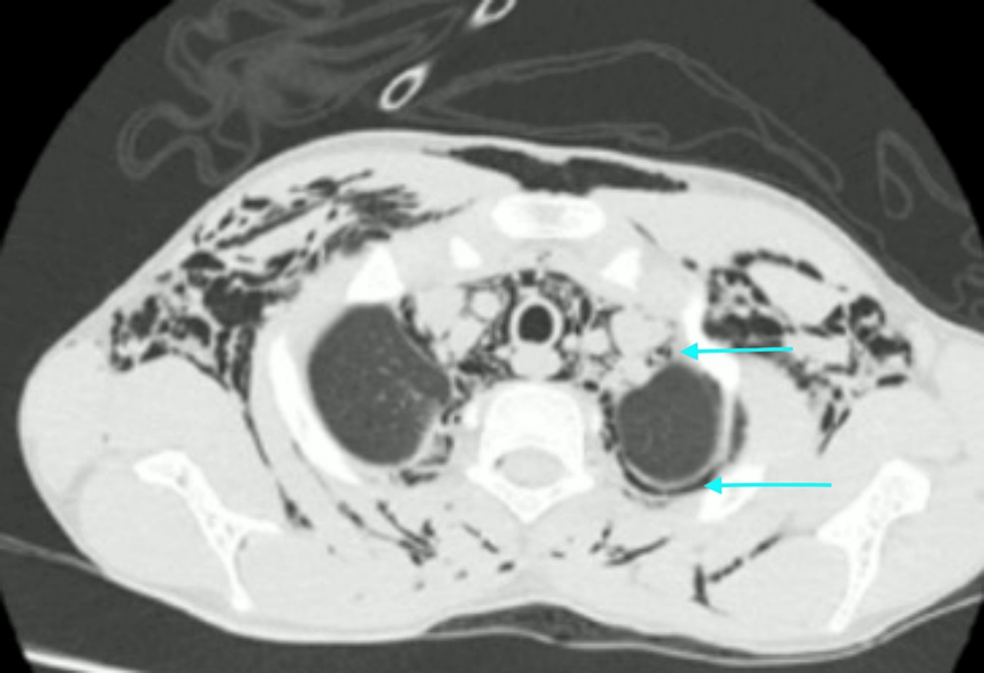
Axial sections through upper chest of HRCT There are tiny apical bullae (upper arrow) and pneumothorax on posterior aspect of left upper chest (lower arrow)

**Figure 6:**
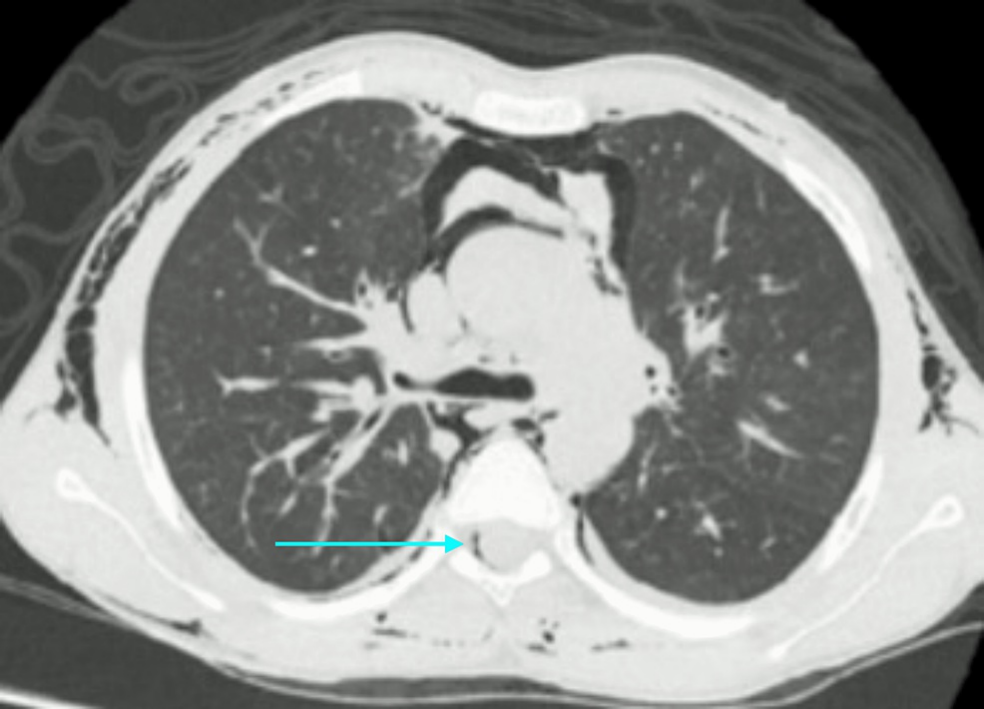
HRCT axial section through the level of carina Note the presence of epidural air (epidural pneumatosis)

HRCT also demonstrated pneumomediastinum and pneumopericardium (Figure 4). There was a small left posterior apical pneumothorax with few minute bullae on left anterior and mediastinal pleura (Figure 5). A thin layer of epidural air was also noted in the cervical spinal canal (epidural pneumatosis) which is a significant finding (Figure 6).

 

**Figure 7:**
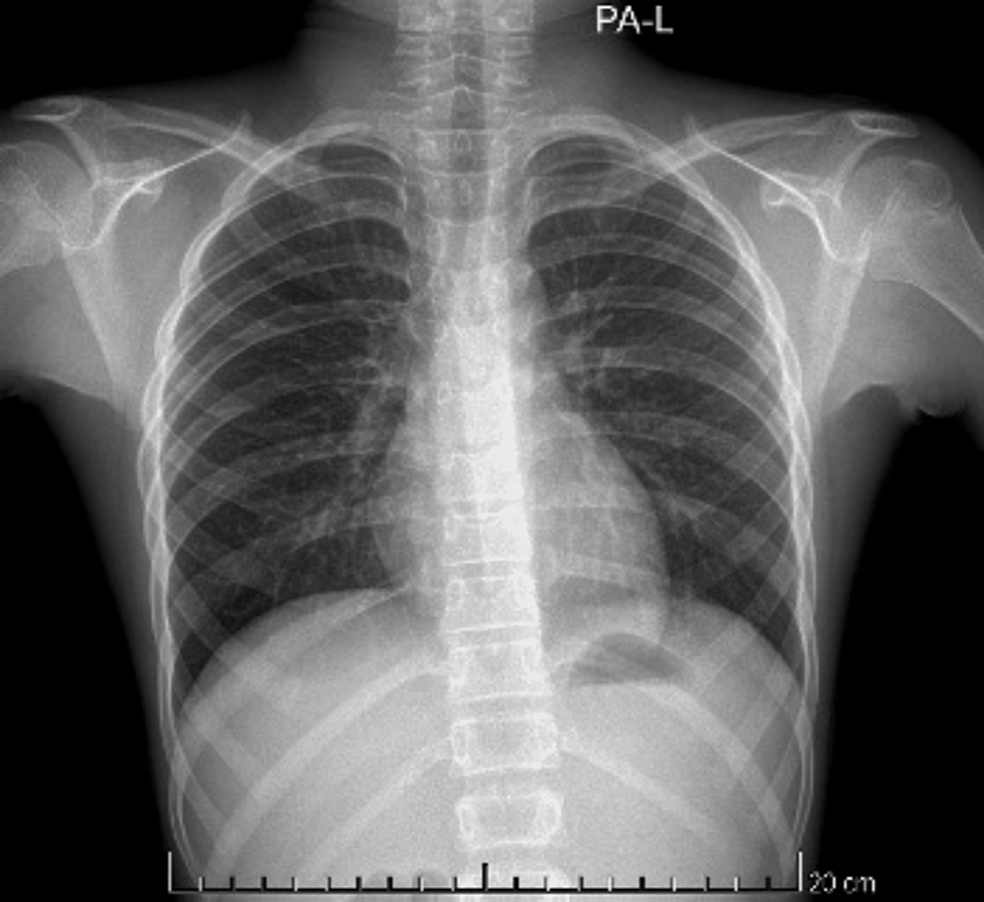
Follow up chest radiograph Radiograph taken two weeks after discharge shows complete resolution of previously noted air collections

**Figure 8:**
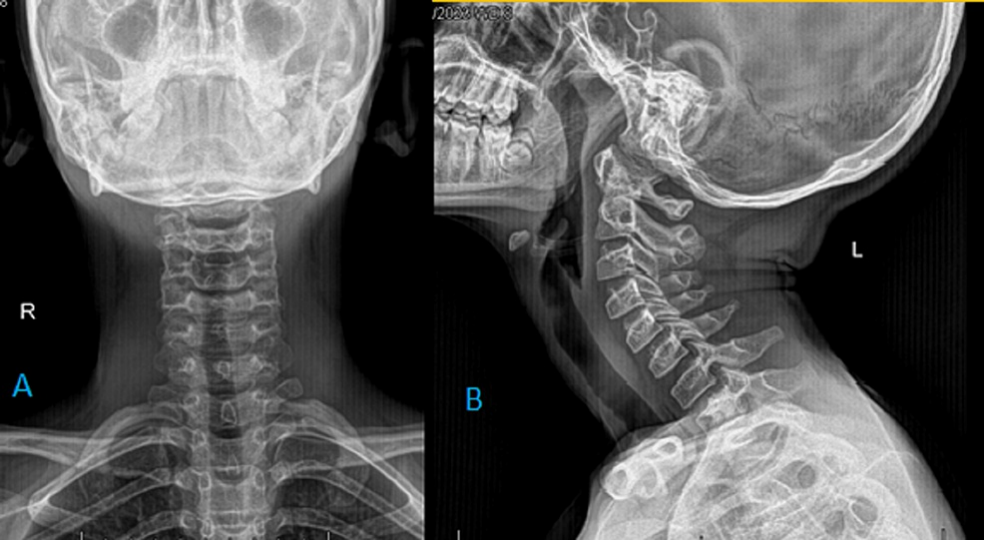
Follow up radiographs of neck AP (A) and lateral (B) views Follow up  radiographs of the neck obtained two weeks after discharge confirms complete resolution of air lucencies

Throughout the in-ward stay of seven days, the patient was hemodynamically stable without any episodes of oxygen desaturation. He was given analgesics for the chest pain and the symptoms improved gradually. By the time he was discharged on the eighth day, he had only a mild chest pain. During the ward review after two weeks of discharge, the patient was completely free of symptoms. The x-ray chest PA (Figure 7) and neck (Figures 8A, 8B) radiographs showed complete radiological resolution of gas lucencies.

## Discussion

SSE and SPM are uncommonly found entities related to abnormal accumulation of air in an anatomical location [10]. This mainly occurs in children and young adults. The reported incidence varies widely, ranging from one in 800 to one in 42,000 in adult and pediatric populations admitted to a hospital [11]. Many more cases may be detected if patients presenting with sudden onset chest pain or shortness of breath are routinely screened for SPM. In young adults admitted for unexplained chest pain or dyspnea, the incidence of SPM was 1:368 [12].

SSE and SPM occur when air leaks through small alveolar ruptures to the surrounding broncho-vascular sheath [ 13]. Since the mean pressure in the mediastinum is always more negative than the pressure in the pulmonary parenchyma, free air tends to move centripetally and spreads into the mediastinum or through the loose mediastinal fascia to the subcutaneous tissues of the chest, neck, and upper extremities [14]. This explains the pathophysiology of radiological findings on plain radiography and HRCT.

Clinical presentation can be varied widely from mild respiratory symptoms to life-threatening acute respiratory events. Sudden onset retrosternal pleuritic type chest pain and dyspnea are the most common presenting symptom but can also present with cough, neck pain, odynophagia, and dysphagia as well [15]. The physical examination can be normal in up to 30% of patients with uncomplicated SSE and SPM [12]. The finding of crepitus in the neck or upper chest is moderately sensitive and highly specific for SSE [16,17]. The auscultation of the heart may find a characteristic crunching sound synchronous with systole which is known as the “Hamman sign” [17].

A chest radiograph is the only investigation needed in the majority of patients with SSE and SPM. Chest x-ray PA and lateral neck x-ray are usually performed. HRCT is performed when chest x-ray findings are inconclusive, or complications are suspected. Lung function test is contraindicated [15]. In addition to establishing the diagnosis of SSE and SPM, the goals of clinical evaluation are to assess potential triggers like asthma or vomiting.

SSE and SPM take a benign course most of the time. Treatment for uncomplicated SSE and SPM is supportive, consisting of analgesia, rest, and avoidance of maneuvers that increase pulmonary pressure (Valsalva or forced expiration, including spirometry) [15]. Asthma or other underlying lung disease is treated as indicated. Most patients recover without sequelae within a few days, and recurrence is rare.

In most cases, SSE and SPM have spontaneous recovery without complications. Occurrence of complications is also rare and most of them resolve spontaneously. Occasionally, air leaks into the abdominal cavity, resulting in pneumoperitoneum [18]. Very rarely, an abrupt rise in mediastinal pressure especially in severe cases can end up with tension pneumomediastinum or tension pneumopericardium. These mainly occur in the setting of mechanical ventilation, with or without thoracic trauma [15]. Air may also leak into the spinal canal, a phenomenon known as pneumorrhachis or pneumotosis this may have associated neurologic signs such as radicular pain and paraplegia [19], which are usually mild and self-limiting.

## Conclusions

SSE with extension into cervical, parapharyngeal, and retropharyngeal spaces, intermuscular air, pneumomediastinum, pneumopericardium, pneumothorax, and epidural pneumatosis as seen in our case, is an extremely rare entity. It is quite important for clinicians to be aware of this condition and know the potential routes of extension and possible complications. Clinical suspicion is essential to plan appropriate investigations especially radiological modalities such as chest x-ray and HRCT. HRCT is especially useful if there is a high clinical suspicion in spite of negative chest findings. This will help in evaluating the severity of the condition, exclude possible etiologies, and look for potential complications so that proper management and follow-up can be planned. Management of SSE and SPM is mostly conservative with pain relief and observation for complications.
